# MOMC-1. Employing the Zika Virus to kill paediatric nervous system tumour cells

**DOI:** 10.1093/noajnl/vdab070.011

**Published:** 2021-07-05

**Authors:** Matthew Sherwood, Robert Ewing, Carolini Kaid, Thiago Giove Mitsugi, Keith Okamoto

**Affiliations:** 1 University of Southampton, Southampton, UK; 2 University of Sao Paulo, Sao Paulo, Brazil

## Abstract

Malignant paediatric nervous system tumours, such as Medulloblastoma, Neuroblastoma and ATRT commonly harbour tumour cells with stem-like features which are highly tumorigenic and resistant to conventional cancer therapies. These tumours can exhibit high lethality and may result in severe sequelae, including cognitive and motor deficits that significantly affect patients’ quality of life. Oncolytic virotherapy is a novel therapy class that exploits viruses that preferentially infect and destroy tumour cells. These viruses present a unique advantage in targeting highly heterogeneous cancers, such as nervous system tumours, as they possess a secondary mechanism of action through which they induce a tumour-specific immune response. Clinical studies employing oncolytic virotherapy have in general reported low toxicity and minimal adverse effects, deeming oncolytic virotherapy as a potentially attractive and safer intervention against paediatric tumours.

The Zika virus (ZIKV) is capable of infecting and destroying neural stem-like cancer cells from human embryonal Central Nervous System (CNS) tumours in vitro and in vivo. Infection of CNS tumour cells with ZIKV effectively inhibits tumour metastasis in mice and, in some cases, induces complete tumour remission. Neuroblastoma arises from immature nerve cells and multiple Neuroblastoma cell lines are susceptible to ZIKV infection and oncolysis. These initial findings have demonstrated the potential for a ZIKV-based virotherapy against paediatric nervous system tumours and warrants examination into the molecular mechanisms through which ZIKV executes its oncolytic ability. My research goal is to elucidate the mechanisms which are of paramount importance for ZIKV-induced oncolysis of brain tumour and Neuroblastoma cells. Utilising global expression omics profiling of ZIKV infection and mapping of viral protein-host protein interactions will identify these mechanisms both at the cellular pathway and molecular levels. These collectively will inform our understanding of how we can employ a future ZIKV-based virotherapy against paediatric nervous system tumours.

